# Urban-Rural Differences in Geodetic Distance Between Home and the Nearest Pharmacy Among Older Pennsylvania Adults

**DOI:** 10.1093/geroni/igaa057.648

**Published:** 2020-12-16

**Authors:** Shivani Khan, Debra Heller, Leroy Latty, Michelle LaSure, Theresa Brown

**Affiliations:** 1 Magellan Rx Management/PACE, Harrisburg, Pennsylvania, United States; 2 Pennsylvania Department of Aging/The PACE Program, Harrisburg, Pennsylvania, United States

## Abstract

Aim: We examined differences in geodetic or straight line distance between home and the nearest community pharmacy among rural and urban older adults in Pennsylvania. Method: The addresses of 241,398 older adults (≥65 years) and 2,880 community pharmacies enrolled in Pennsylvania’s Pharmaceutical Assistance Contract for the Elderly (PACE) program in 2018 were geocoded. We identified pharmacies in the same or adjacent counties for each enrollee and measured the geodetic distance between home and those pharmacies. The pharmacy with the shortest distance from home was identified as the nearest pharmacy for each enrollee. Enrollees’ home addresses were categorized as urban or rural at the county level, based on the Center for Rural Pennsylvania’s definitions. T-tests and chi-squared tests were used for analyses. Results: Overall, 37% were rural older adults and the mean distance between home and the nearest pharmacy was 1.60 ± 2.21 miles. The mean distance between home and the nearest pharmacy was significantly greater in rural compared to urban older adults (2.78 ± 2.93 versus 0.91 ± 1.19; p<.0001). A higher proportion of rural older adults resided >5 miles away from the nearest pharmacy compared to urban older adults (19.19% versus 1.80%; p<.0001). Moreover, 2.96% of rural older adults resided >10 miles away from the nearest pharmacy compared to 0.08% of urban older adults (p<.0001). Conclusion: Older patients in rural counties need to travel longer distances for pharmacy access than in urban counties. Efforts to provide convenient access to medications and pharmacy services for rural older patients are necessary.

